# Year-long, multiple-timepoint field studies show the importance of spatiotemporal dynamics and microbial functions in agricultural soil microbiomes

**DOI:** 10.1128/msystems.00112-25

**Published:** 2025-07-02

**Authors:** Lisa Joos, Sarah Ommeslag, Steve Baeyen, Wouter Asselberg, Koen Van Loo, Lieven Clement, Jane Debode, Bart Vandecasteele, Caroline De Tender

**Affiliations:** 1 Department of Biochemistry and Microbiology, Ghent University 26656, Ghent, Belgium; 2 Flanders Research Institute for Agriculture, Fisheries and Food (ILVO), Plant Sciences Unit, Merelbeke, Belgium; 3 Department of Applied Mathematics, Computer Science and Statistics, Ghent University 366743 , Ghent, Belgium; University of California, Davis, Davis, California, USA

**Keywords:** agriculture, biochar, compost, metabarcoding, metatranscriptomics, PLFA, soil microbiome, spatiotemporal dynamics

## Abstract

Despite the recognition of the complexity of soil ecosystem dynamics, most soil microbiome studies sample one field, take one sample per field, or use limited samples throughout the year. This limits our understanding of the spatiotemporal role of the soil microbiome in relation to management practices. To address these limitations, we conducted a year-long investigation of the soil microbiome in two agricultural fields, sampling multiple plots at different soil depths every 5 weeks. We examined spatial and temporal variabilities in response to the application of organic amendments (one-time biochar and annual compost application) on bacterial and fungal communities, studying both the microbial composition (metabarcoding, phospholipid fatty acids [PLFA], hot-water extractable-carbon) and activity (metatranscriptomics). Indicated by metabarcoding and PLFA, fungal communities were less affected over time per field, whereas bacteria exhibited more pronounced temporal trends. In contrast, fungi displayed clear spatial effects, while bacterial spatial differences within the field were predominantly observed in the deeper soil layer. Effects on functional roles and metabolic processes of the active microbial community were mainly related to temporal trends, especially in the topsoil. Organic amendments did not affect the microbial activity and affected fewer than 2% of the bacterial and fungal amplicon sequence variants over time. This study reveals the predominance of spatiotemporal dynamics over management practices in shaping soil microbial communities within agricultural fields, emphasizing the importance of field-specific factors, sampling depth, and community type. This ushers in the need for a well-considered experimental design and sampling strategy that accounts for spatiotemporal trends.

**IMPORTANCE:**

This study addresses a critical gap in soil microbiome research by investigating spatiotemporal effects on soil bacterial and fungal composition and activity in relation to field management practices. Moving beyond single-field and limited sampling approaches, this research conducted monthly sampling events on two fields at various depths. By combining metabarcoding, phospholipid fatty acid analysis, and metatranscriptomics, the study examined bacterial and fungal community composition, biomass, and functionality. Key findings reveal distinct responses of bacterial and fungal communities to spatiotemporal variability and management practices. Functional categories were predominantly driven by temporal trends rather than compost amendments. Temporal changes were more pronounced in the topsoil. These insights into the complex interactions between soil microbial communities, management practices, and spatiotemporal dynamics contribute significantly to soil microbiome research and sampling strategies.

## INTRODUCTION

Microbial communities in agricultural fields are exposed to a wide variety of environmental changes, which can elicit specific responses in the soil microbiome (1). Temporal changes in environmental conditions, such as soil temperature, moisture, and nutrient content, can strongly impact microbial activity (2), biomass (3, 4), diversity (5), and community composition (6–8). Moreover, soils are heterogeneous, as they consist of unevenly distributed components, such as minerals, organic matter, water, air, and living organisms (9). These spatial variations can mask temporal effects in natural systems (8). For agricultural soils, temporal shifts are hypothesized to surpass spatial effects, as these types of soils tend to be more homogenous than natural systems due to extensive management practices (10).

Microbial communities can also vary with depth; topsoil typically has greater microbial biomass and diversity than deeper layers do, which are dominated by prokaryotes (11, 12). The topsoil layer is also influenced the most by soil management and temporal effects.

Whereas seasonal and spatial variations are hypothesized to have the greatest impact on the diversity and community structure of microorganisms in soil, management practices, such as organic amendments, can also have significant effects (2, 4, 12). Studies have reported an increase in the microbial biomass after compost amendments compared to mineral fertilization (13, 14). Long-term shifts in the microbial community have also been observed in response to annual compost application (15–17). Compost amendments resulted in a small number of relative enrichments of Bacteroidetes and Chloroflexi and depletions of the Proteobacterial and Acidobacterial families over time (14). These changes have mainly been found to cause rather gradual effects (18). Therefore, we hypothesize that the addition of organic amendments has a lower impact than spatiotemporal trends do and that the amendment effect might differ depending on the sampling location and/or season.

While the above studies rely primarily on metabarcoding and taxonomic analyses to study microbial communities, advancements in sequencing technologies are now facilitating the exploration of microbial functionality. Metatranscriptomics, a technique that sequences total RNA from environmental samples, has emerged as a powerful tool for studying the active microbial community.

By targeting total RNA, this approach provides insights into the metabolically active microorganisms and their potential functional roles within the soil ecosystem. Studying the active community through metatranscriptomics can enhance our understanding of the temporal dynamics in the soil and how organic amendments alter the soil microbiome. However, few studies, especially in the agricultural context, have investigated the temporal trends in the functionality of the active community. In a study of rhizosphere grasslands, a strong temporal driver was found for specific gene pathways, with a shift from maintenance metabolism in winter to growth during summer (19). A similar conclusion was reached for the topsoil of a coniferous forest, where winter was found to be a resting period, and summer, a period of active metabolism based on metabolic pathways (20). The impact of compost amendment on the functionality of the soil microbiome in combination with temporal trends has not yet been studied in field trials. However, studies have revealed upregulation of genes related to N cycling and a strong increase in enzymatic activity (21–23). Overall, we hypothesize to observe more functional shifts related to compost application than seasonal changes.

In this study, we aimed to elucidate the spatiotemporal dynamics of the soil microbiome in two agricultural fields in relation to organic amendments (compost and biochar) as sources of stable carbon. Stable carbon is here defined as carbon that breaks down slowly and contributes to long-term carbon accumulation in soil, leading to increased carbon storage. We hypothesized that: (i) microbial shifts (either taxonomical or functional) are influenced mostly by temporal changes, followed by within-field heterogeneity and organic amendments; (ii) organic amendments will have a more pronounced role on microbial functions compared to taxonomical shifts; and (iii) fewer microbial shifts in taxonomy will be observed in the deeper layer than in the top layer over time.

## RESULTS

### Bacterial and fungal communities respond differently to spatiotemporal variability in the topsoil layer

The permutational multivariate analyses of variance (PERMANOVA) showed no significant interaction between the factors ‘time’ and ‘treatment’ in any field (PERMANOVA, *R*^2^ < 0.1, *P* > 0.05) (File S1). The microbial community composition changed significantly over time in both fields, explaining 30% of the variation (PERMANOVA, *R*^2^ > 0.30, *P* < 0.001), whereas the effect of treatment was statistically significant but only explained 2% of the variation (PERMANOVA, *R*^2^ = 0.02, *P*_treatment_ = 0.006). The spatial variability across field blocks within Field B had a small, statistically significant effect on the bacterial community, explaining 5% of the variation (PERMANOVA, *R*^2^ = 0.05, *P* < 0.001), and a larger effect on the fungal community, explaining 10% of the variation (PERMANOVA, *R*^2^ = 0.10, *P* < 0.001) (File S1).

**Fig 1:**
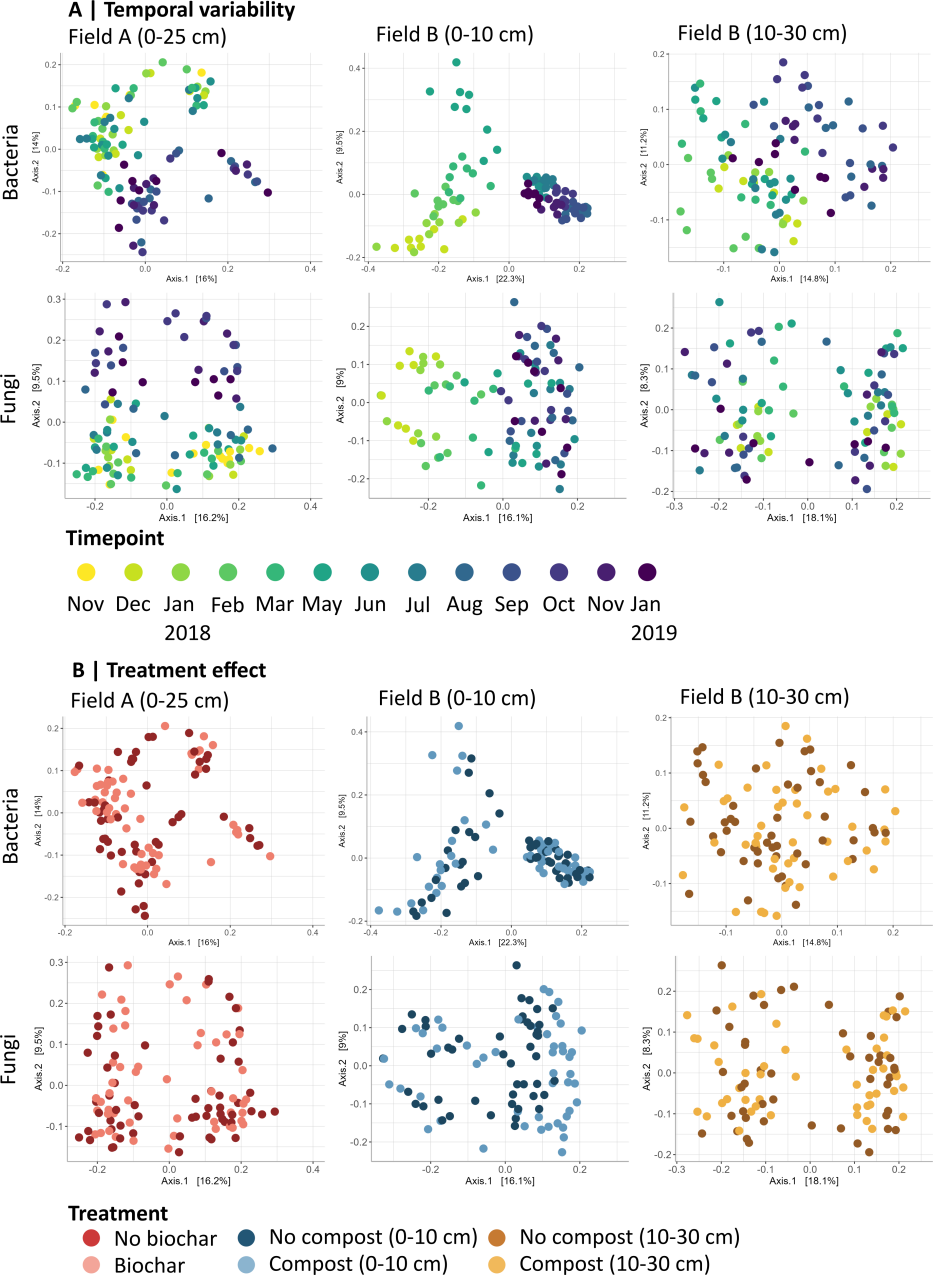
Principal coordinate analysis plots showing (**A**) temporal and (**B**) treatment effects on bacterial and fungal ASV communities. In all graphs, a Bray-Curtis dissimilarity matrix was calculated for the bacterial and fungal amplicon sequence variant data across all timepoints (nFieldA = 13 and nFieldB = 12). The percentage of variation indicated on each axis represents the fraction of total variation explained in the dataset. Within each section, the effects of (**A**) temporal variability and (**B**) treatment are shown. For (**A**) temporal variability, a range of colors is utilized, starting from yellow for the first sampling month and progressing to green and purple for the subsequent sampling months. For each timepoint and treatment combination, there were four biological replicates. For the (**B**) treatment, the colors indicate the two fields and applied amendments. Field A is indicated by red, Field B 0-10 cm by blue, and Field B 10-30 cm by brown. Amendments within each field are depicted using two shades; light shades denote non-amended samples, and dark shades are amended samples with either biochar (Field A) or compost (Field B).

**Fig 2:**
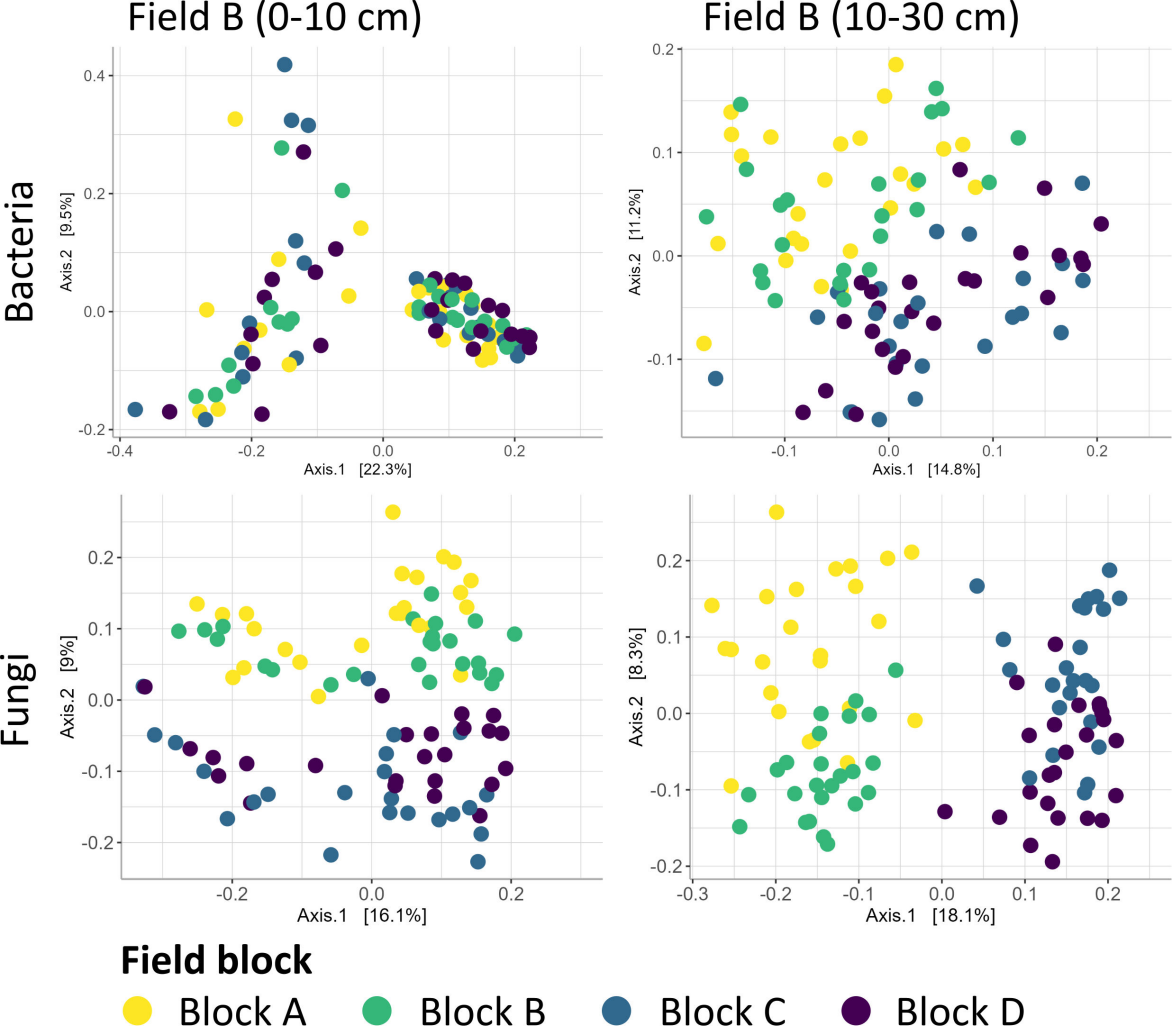
Principal coordinate analysis plot displaying within-field variability on bacterial and fungal ASV communities. A Bray-Curtis dissimilarity matrix was calculated for the bacterial and fungal ASV data across all timepoints (n_FieldA_ = 13 and n_FieldB_ = 12). The percentage of variation indicated on each axis represents the fraction of total variation explained in the dataset. The colors are assigned to different field blocks of Field B. Block A is depicted in yellow, Block B in green, Block C in blue, and Block D in purple. For each timepoint and treatment combination, there are four biological replicates.

Temporal, treatment, and spatial effects on microbial community composition were visualized through principal coordinate analysis (PCoA) plots, with bacterial and fungal communities clustered by temporal changes along the second axis in Field A and the first axis in Field B (Fig. 1A). In contrast, the treatment effects were less pronounced and not as clearly distinguished in the PCoA (Fig. 1B). The fungal PCoA for the spatial variability showed distinct spatial separation between blocks AB and CD, with clustering along the *y*-axis in the top layer (9% of the variation), while this was not the case for the bacterial community in the top layer (Fig. 2).

These shifts were not reflected in the alpha-diversity, where no changes occurred in time, space, or by treatment (Fig. S1).

To gain further insights into the primary drivers of the soil bacterial and fungal community structure, differential analyses were conducted at the amplicon sequence variant (ASV) level. Specifically, we examined the following drivers on the microbial composition: (i) the effect of treatment (amended vs. non-amended) within each timepoint, (ii) identifying differences among various timepoints within the non-amended soils, with particular emphasis on seasonal comparison, the heatwave period, and 1 year apart, and (iii) the spatial variability in the non-amended Field B blocks.

**Fig 3:**
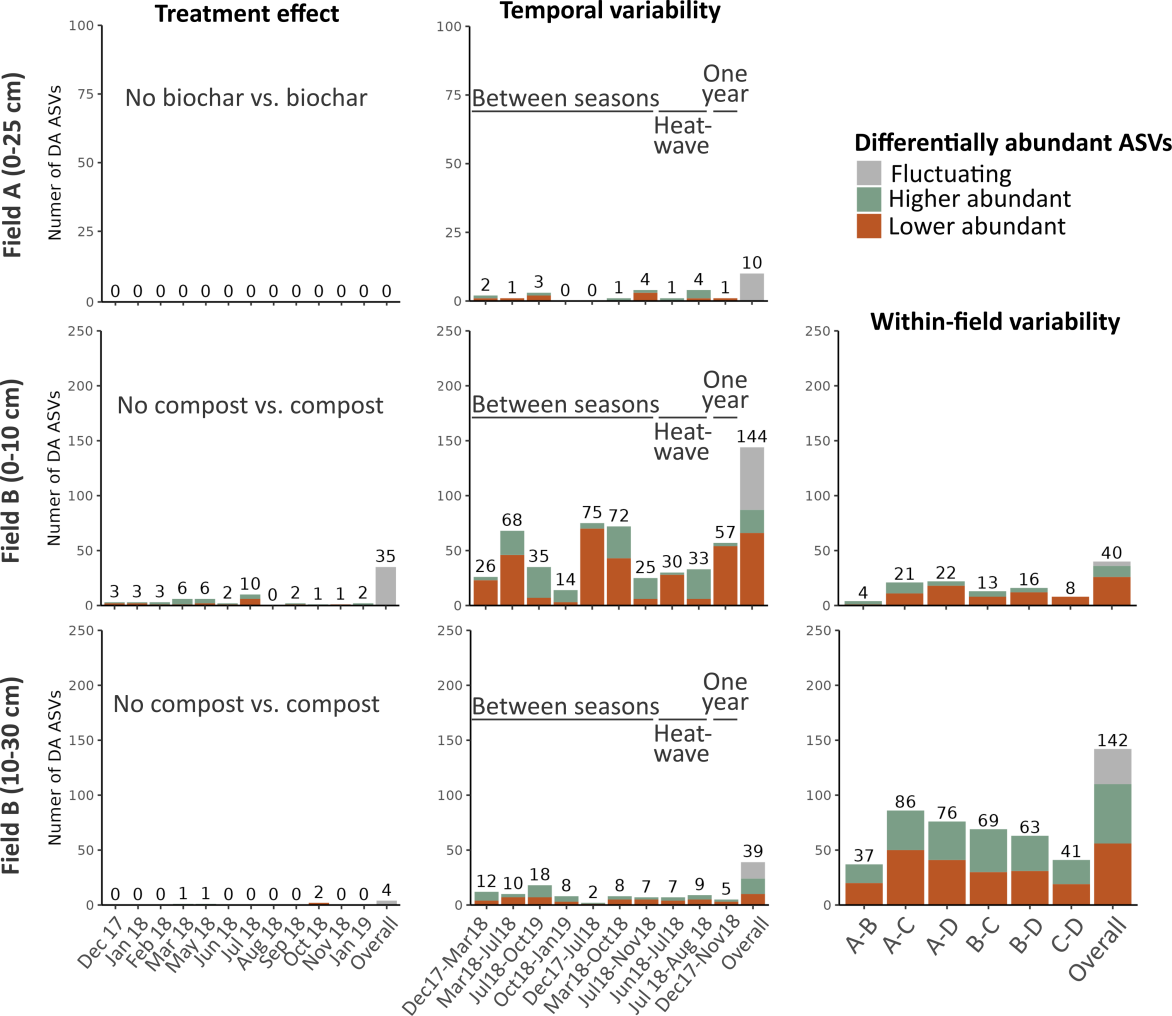
Differential abundance analyses of bacterial amplicon sequence variants (ASVs) for the treatment and temporal effects and within-field variability. The stacked bar plots show the number of differentially abundant (DA) ASVs on a 5% false discovery rate level using a stage-wise approach. Three analyses were conducted, namely, (i) the treatment effects (amended vs. non-amended) within each timepoint, (ii) the differences between various timepoints in non-amended soil, and (iii) the within-field heterogeneity by comparing the non-amended field blocks with each other (only in Field B). Within each of these analyses, an omnibus hypothesis was conducted, and DA ASVs in at least one hypothesis are included as “overall.” Bars are colored to show the proportion of ASVs that had an increase or decrease in relative abundance. Overall, DA ASVs that significantly increased or decreased throughout the different hypotheses are labeled as “fluctuating.”

**Fig 4:**
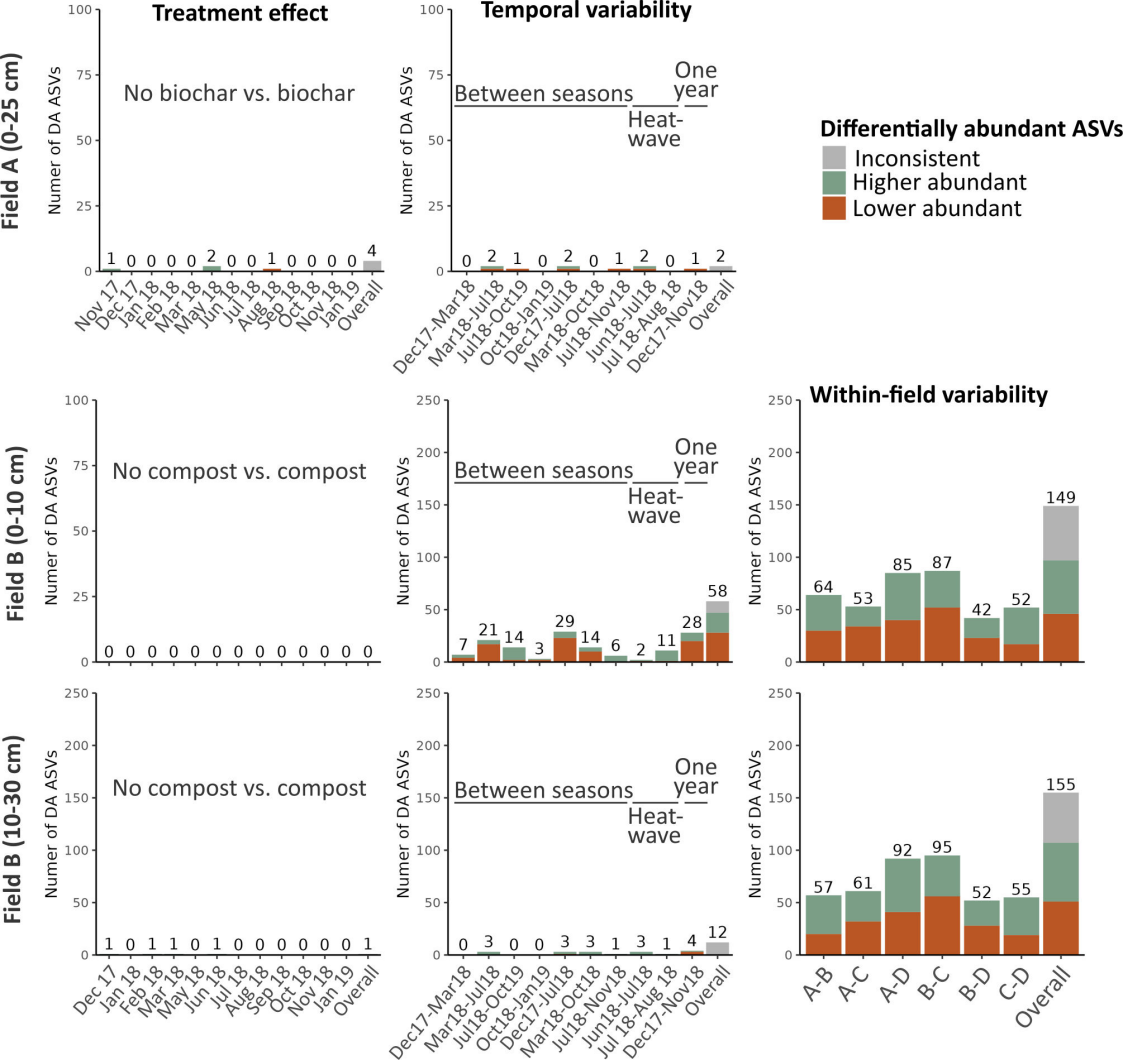
Differential abundance analyses of fungal amplicon sequence variants (ASVs) for the treatment and temporal effects and within-field variability. The stacked bar plots show the number of differentially abundant (DA) ASVs on a 5% false discovery rate level using a stage-wise approach. Three analyses were conducted, namely, (i) the treatment effects (amended vs. non-amended) within each timepoint, (ii) the differences between various timepoints in non-amend soil, and (iii) the within-field heterogeneity by comparing the non-amended field blocks with each other (only in Field B). Within each of these analyses, an omnibus hypothesis was conducted, and DA ASVs in at least one hypothesis are included as “overall.” Bars are colored to show the proportion of ASVs that had an increase or decrease in relative abundance. Overall, the DA ASVs that significantly increased or decreased throughout the different hypotheses are labeled as “fluctuating.”

First, to study the effect of treatment, we found that in Field A, the biochar treatment elicited a response of merely 1% of the total bacterial and fungal ASVs. A similar response was observed in the analysis of the different timepoints, where a response was detected in less than 2% of the bacterial ASVs and 0.4% of the fungal ASVs (Fig. 3 and 4). Similar for Field B, almost no shifts in ASVs were noted after compost treatment (Fig. 3).

Second, we observed that the temporal variability in the top layer of Field B was the primary driver for shifts in the bacterial community, accounting for 21.0% of the total bacterial ASVs. The highest number of differentially abundant bacterial ASVs is observed between winter vs. summer, spring vs. summer, and spring vs. autumn (Fig. 3). Conversely, the heatwave led to fewer differentially abundant bacterial ASVs. Notably, even after the passage of 1 year, a substantial number of differential abundant bacterial ASVs persist. For the fungal community in the top layer of Field B, a weaker temporal effect was observed, affecting only 15.5% fungal ASVs (Fig. 4). In contrast, spatial variability and treatment effects had relatively small effects on the bacterial community, resulting in only 5.8 and 5.1% differential abundant bacterial ASVs, respectively (Fig. 3).

Third, when comparing the various blocks, the A vs. B and C vs. D comparisons show the least number of differentially abundant (DA) bacterial ASVs (Fig. 3). The fungal ASVs, however, had the highest number of DA ASVs in the blocks that are across from each other, with 39.9% differential abundant fungal ASVs (Fig. 4).

**Fig 5:**
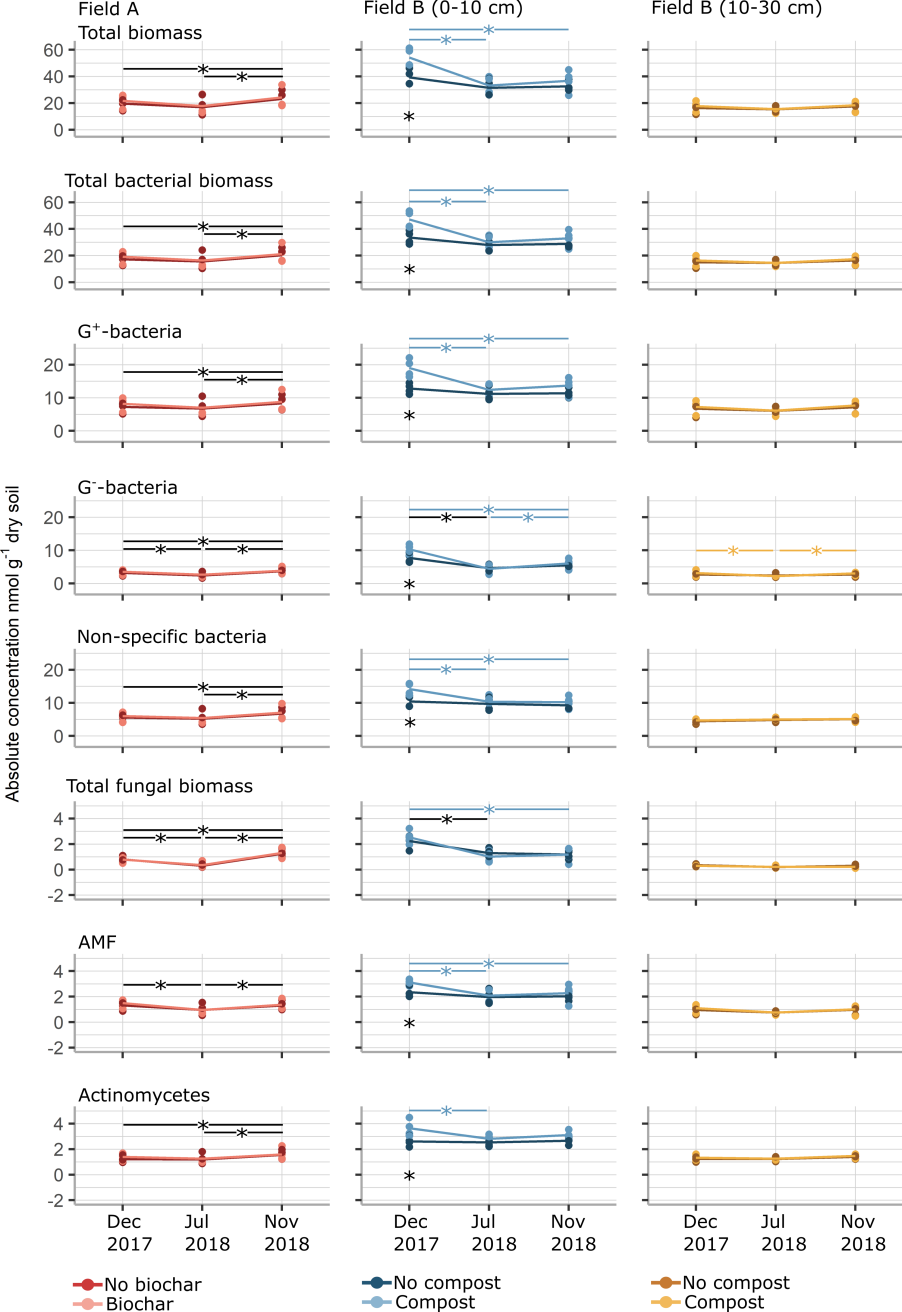
Absolute concentrations (nmol g^−1^ dry soil) of all PLFA groups and combinations (total biomass, total bacteria, and total fungi) for three timepoints in fields A and B. The different treatments within fields and depths are indicated by color. The four biological replicates are represented by dots, and the mean is depicted by a line. A black asterisk (*) beneath the line graphs indicates a significant difference between treatments within a timepoint. The line and the asterisk (*) denote significant differences between timepoints, where a black line indicates that both treatments were significant, while a colored line indicates a significant difference in only one treatment (GLM; *P* < 0.05).

Compared with the metabarcoding data, the phospholipid fatty acids (PLFAs) had a similar prominent temporal effect on the microbial community. The PLFA concentration decreased toward the summer of July 2018 and subsequently increased in the winter of November 2018 (Fig. 5). However, this effect was significant mainly in the top layer of Field B, where compost amendment resulted in increased biomass during the initial winter period (GLM, *P* < 0.05).

**Fig 6:**
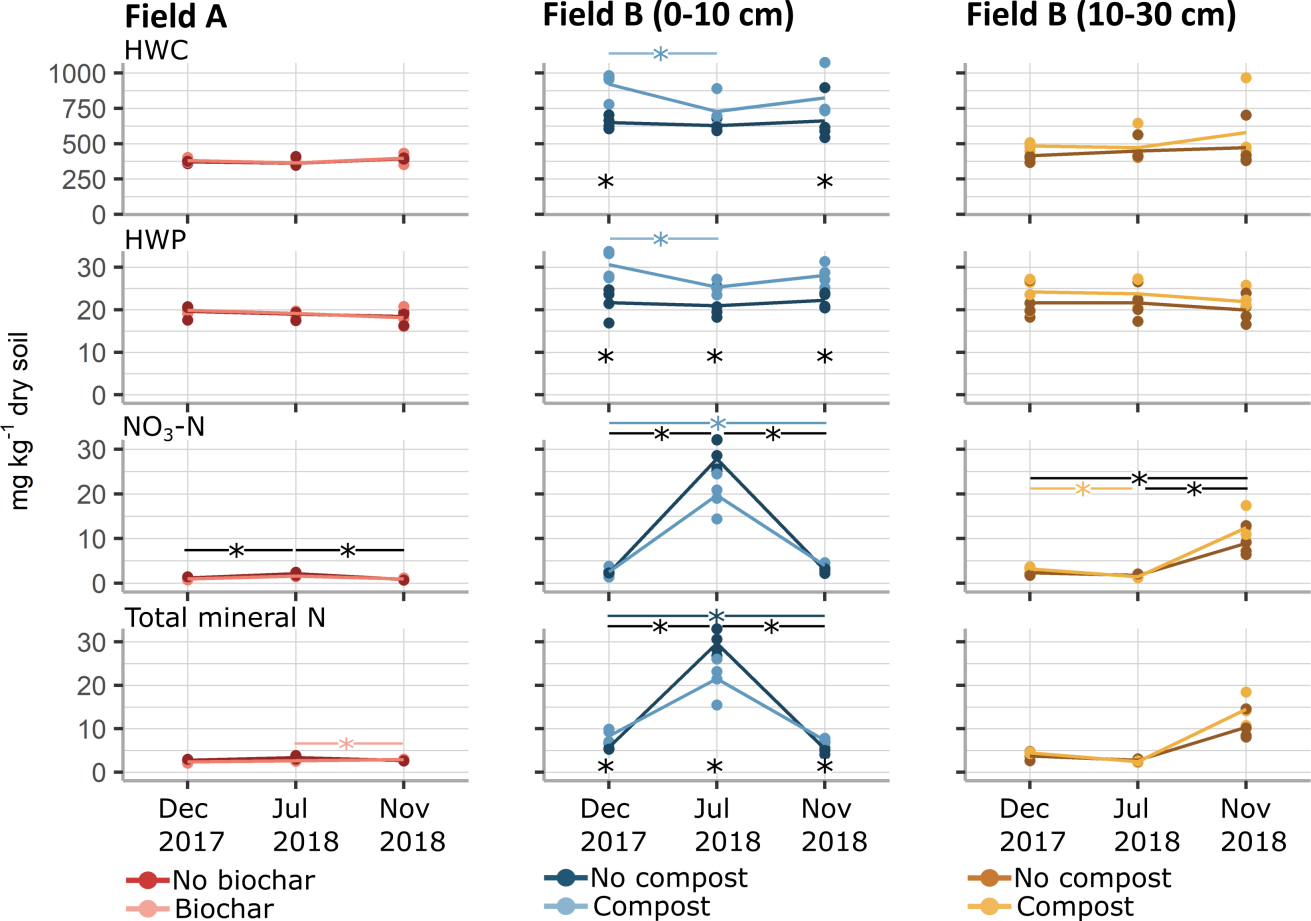
Concentrations of hot water extractable-carbon (HWC), hot water extractable-phosphorus (HWP), NO_3_-N, and total mineral N in fields A and B. The colors indicate the fields, treatments, and depths. The four biological replicates are depicted by the dots, and the line reflects the mean. The and asterisk (*) denote significant differences between timepoints, where a black line indicates that both treatments were significant, while a colored line indicates a significant difference in only one treatment (GLM; *P* < 0.05).

Within the top layer of Field B, a significant surge in NO_3_-N was accompanied by a decline in NH_4_-N as summer approached, which was also visible to a lesser extent in Field A (GLM, *P* < 0.05, Fig. 6). Furthermore, hot water extractable-carbon (HWC) concentration in the two winter periods and throughout the three timepoints for hot water extractable-phosphorus (HWP) were significantly lower in the controls than in those with compost in Field B (GLM, *P* < 0.001, Fig. 6).

### Temporal dynamics shape microbial functions, not organic amendments

To study changes in microbial activity over time in relation to the addition of organic amendments (compost), the expressed microbial genes were measured for Field B via metatranscriptomics on three timepoints: two winter periods (December 2017, November 2018) and one summer period (July 2018), which were chosen because of significant fluctuations in soil moisture between these time periods (Fig. S2). The MG-RAST analysis revealed that 18% of the merged and filtered sequences were annotated to a protein and classified into 926 functional categories, level 3.

**Fig 7:**
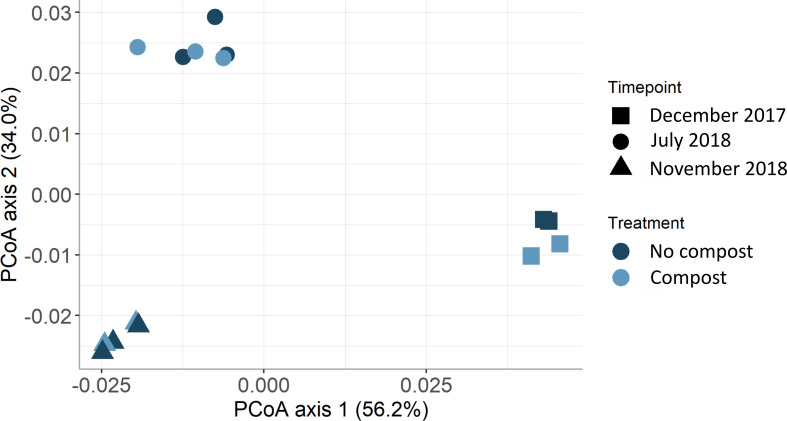
Principal coordinate analysis plot of the MG-RAST subsystem level 1 in Field B. PCoA shows three different timepoints (December 2017, July 2018, and November 2018) and compost effect in Field B. Color shading depicts the treatment (light = non-amended, dark = compost-amended). The symbols indicate three different timepoints.

The PERMANOVA results indicated no significant interaction between the factors ‘treatment’ and ‘time point’ (PERMANOVA, *R*^2^ = 0.76, *P*_treat:time_ = 0.3). The impact of temporal variability had the greatest effect (PERMANOVA, *R*^2^ = 0.92, *P*_time_ = 0.001) compared to the absent effect of organic amendment on these functional categories (PERMANOVA, *R*^2^ = 0.01, *P*_treat_ = 0.25). In particular, there was a large difference in functional categories between the two winter periods vs. summer period based on the PCoA plot, accounting for more than 73.1% of the total variability of the dataset (Fig. 7).

While changes in the microbial community composition were rather limited over the three points, the differential expression of genes changed drastically. Among the 926 functional categories in the SEED subsystems level 3 determined by MG-RAST, 56.3% were differentially expressed at the 5% FDR level between the first winter and summer periods, and 70.8% were differentially expressed between the summer and second winter periods. The differences between the two winter periods were less pronounced, as 47.1% of the functional categories were significantly expressed at the 5% FDR level. Remarkably, in both comparisons (first winter-summer and summer-second winter), there were more functional categories downregulated compared to upregulated genes (Fig. S3). In the remainder of this paper, we focus on the subset of these differentially expressed functional categories level 3 with an absolute logFC of at least 2. This left 56 functional categories for the first winter-summer period, 65 for summer-second winter period, and only 10 between the two winter periods (Table 1).

**TABLE 1:** Functional categories of MG-RAST, which were significantly up- or downregulated between three time comparisons^*a*^

Level 1	Level 2	Level 3	Dec. 17	July 18	Nov. 18
Amino acids and derivatives	Aromatic amino acids and derivatives	Indole pyruvate oxidoreductase complex	0.07	0	0.18
Carbohydrates	CO_2_ fixation	CO_2_ uptake, carboxysome	0.6	**3.65**	0.86
	Monosaccharides	L-ascorbate utilization (and related gene clusters)	0.03	0.01	0.05
	NULL	VC0266	0.05	0.01	0.07
	One-carbon metabolism	Methanogenesis from methylated compounds	0.1	0	0.17
	Polysaccharides	Xyloglucan utilization	0.01	**0.07**	0.03
Cell wall and capsule	Capsular and extracellular polysaccharides	Extracellular polysaccharide biosynthesis of streptococci	0.06	0.01	0.15
		Xanthan exopolysaccharide biosynthesis and export	0.06	0	0.07
Clustering-based subsystems	CRISPRs and associated hypotheticals	CBSS 216592 1 peg 3534	0.03	0.01	0.08
	DNA metabolism	CBSS 269801 1 peg 2186	0.06	0.01	0.05
	NULL	CBSS 176280 1 peg 1561	2.2	**12.21**	2.54
		Glutaredoxin 3 containing cluster 2	0.24	**0.93**	0.17
		USS DB 7	2.56	0.45	6.16
Cofactors, vitamins, prosthetic groups, pigments	Riboflavin, FMN, FAD	Flavodoxin	0.34	**1.42**	0.5
Dormancy and sporulation	NULL	Spore sore dehydration	0.08	0.01	0.1
Iron acquisition and metabolism	NULL	ABC transporter [iron] B12 [siderophore hemin]	0.03	0.01	0.08
Membrane transport	ABC transporters	ABC transporter peptide (TC3A155)	0.11	0.01	0.08
	Protein secretion system, Type III	Type III secretion system	0.07	0.01	0.11
		Type III secretion system orphans	0.08	0.01	0.17
Metabolism of aromatic compounds	NULL	Cresol degradation	0.05	0	0.06
	Peripheral pathways for catabolism of aromatic compounds	Phenol hydroxylase	0.09	0.01	0.17
Miscellaneous	NULL	Archease2	0.01	**0.06**	0.04
	Plant-prokaryote DOE project	DOE COG3533	0.04	0	0.11
Nitrogen metabolism	NULL	Dissimilatory nitrite reductase	0.08	0	0.1
Nucleosides and nucleotides	Purines	Xanthine metabolism in bacteria	0.08	0.02	0.19
Phages, prophages, transposable elements, plasmids	Plasmid-related functions	Plasmid-encoded T DNA transfer	0.05	0	0.05
Photosynthesis	Electron transport and photophosphorylation	Photosystem I	0.61	**5.26**	0.4
		**Photosystem II**	3.5	**31.84**	1.48
	Light-harvesting complexes	Chlorosome	0.03	0.02	0.1
Protein metabolism	Protein biosynthesis	Ribosome SSU chloroplast	0.03	**0.12**	0.02
Regulation and cell signaling	Proteolytic pathway	Coagulation cascade	0.04	0.01	0.2
	Quorum sensing and biofilm formation	Quorum sensing regulation in *Pseudomonas*	0.05	0	0.04
Respiration	Electron-accepting reactions	Fe(III) respiration *Shewanella* type	0.03	0	0.14
	Electron-donating reactions	Coenzyme F420 H2 dehydrogenase (methanophenazine)	0.03	0.01	0.06
		**H2:CoM S S HTP oxidoreductase**	0	**0.2**	0.06
	NULL	Biogenesis of cbb3 type cytochrome c oxidases	0.15	0.01	0.12
	NULL	Cytochrome B6 F complex	0.18	**1.59**	0.13
	Reverse electron transport	Membrane bound hydrogenases	0.03	0	0.13
Secondary metabolism	Bacterial cytostatics, differentiation factors and antibiotics	Clavulanic acid biosynthesis	0.01	0.1	0.04
Stress response	Osmotic stress	Ectoine biosynthesis and regulation	0.09	**0.59**	0.09
	Oxidative stress	CoA disulfide thiol disulfide redox system	0.05	0	0.08
Sulfur metabolism	NULL	Sulfate reduction associated complexes	0.04	0	0.08
Virulence, disease, and defense	Bacteriocins, ribosomally synthesized antibacterial peptides	Marinocine, a broad spectrum antibacterial protein	0.04	0	0.07
	Invasion and intracellular resistance	Cytolysin and lipase operon in *Vibrio*	0.07	0	0.05
		*Listeria* surface proteins: LPXTG motif	0.07	**0.99**	0.19

^*a*^ The relative normalized counts of the functional categories are shown for each time point and color-coded depending on an up- or downregulation during summer compared to the two winter periods (December 2017 and November 2018). Bold numbers for July 2018 indicate all the categories that increase during the summer period compared to the winter period; otherwise, a decrease was observed. The two functional categories in level 3 in bold are the only significant parameters among the three time comparisons.

Among these, 45 level 3 functional categories were differentially expressed in both winter-summer comparisons (Table 1). Only two level 3 functional categories were differentially expressed across the three period comparisons and attributed to an HTP oxidoreductase and photosystem II, both of which presented relatively higher expression in the summer. The most dominant functional categories at level 3 were those involved with clustering-based subsystems, photosynthesis, and carbohydrates throughout all three time points, although large fluctuations were observed. The functional categories level 3 (32/45) were downregulated during the summer period and upregulated again once moisture levels increased. The functional categories level 3 of photosynthesis and CO_2_ uptake were strongly upregulated during summer. An increase in osmotic stress response was also observed during summer but not during dormancy and spore regulation. On the contrary, downregulation was observed for some clustering-based subsystems, metabolism, and transport functionalities during summer.

### Less temporal changes in the deeper soil layer compared to the top layer

The only significant interaction effect observed was between the factors ‘time’ and ‘depth’ for the bacterial and fungal ASVs, indicating that changes over time had different effects on the community dynamics at varying depths (PERMANOVA, *R*^2^ = 0.08, *P*_time:depth_ < 0.001) (File S1). In accordance with the top layer, the bacterial community in the deeper layer was primarily influenced by temporal factors, which accounted for 34% of the variation (PERMANOVA, *R*^2^ = 0.34, *P*_time_ = 0.001). Within-field variability was the second most important factor, explaining 18% of the variation (PERMANOVA, *R*^2^ = 0.18, *P*_block_ = 0.001), followed by a minor significant effect of treatment (PERMANOVA, *R*^2^ = 0.02, *P*_treatment_ = 0.001). For the fungal community, however, the within-field variability and temporal effect in the deeper layer explained the same percentage of variance (PERMANOVA, *R*^2^ = 0.18, *P*_time_ = 0.001), followed by a limited significant effect for treatment (PERMANOVA, *R*^2^ = 0.02, *P*_treatment_ = 0.001). The treatment, temporal, and spatial effects in the deeper layer were also visualized using PCoA (Fig. 1 and 2). Temporal effects were observed for the bacterial community along the *x*-axis (14.8% of the variation) but not for fungi. The bacterial community showed a clear separation between block AB and CD along the *y*-axis (11% of the variation), while the fungal community exhibited distinct clustering across all blocks. This resulted in four separate clusters along both the *x*- (18.1% variation) and *y*-axes (8.3%).

In general, no significant differences in the bacterial and fungal diversities between the top and deeper layers were observed (GLM, *P* > 0.05; Fig. S1).

When differential analyses of the ASV level were performed via a stage-wise approach, within-field variability was found to have the greatest percentage of DA ASVs, accounting for approximately 40% of all bacterial and fungal ASVs (142 bacterial DA and 155 fungal DA ASVs). The temporal effect resulted only in 39 DA bacterial ASVs (11% of all bacterial ASVs) and 12 DA fungal ASVs (3% of all fungal ASVs). Treatment only affected less than 1% of the ASVs (Fig. 3 and 4).

Upon comparing the PLFA concentrations in two soil layers (0–10 and 10–30 cm), a significant difference was observed across all time points, with the deeper layer exhibiting only half the biomass concentration of the top layer (GLM, *P* < 0.001, Fig. 5). For example, in November 2018, the total biomass in the untreated samples was 39.3 ± 2.9 nmol g^−1^ DM in the top layer and only 16.3 ± 1.7 nmol g^−1^ DM in the deeper layer. In general, no significant time or treatment effect was observed in the deeper layer in the PLFA groups in contrast (GLM, *P* > 0.05) to what was observed in the top layer (see above). Only the G^−^ bacteria had a small significant decrease toward the summer (GLM, *P* = 0.023) and increase towards the winter of 2018 (GLM, *P* = 0.052).

## DISCUSSION

### Bacterial and fungal communities respond differently to spatiotemporal variability

We hypothesized that the strongest impact on the soil bacterial and fungal communities would come from the temporal effect, followed by the spatial and treatment effects. However, we discovered that the spatiotemporal effect was dependent on the field, sampling depth, and studied community. This highlights the complexity of the soil environment and microbial communities and the need for the right sampling time (8). While we observed temporal effects on the microbial community in Field A, more pronounced fluctuations in bacterial communities were evident in the top layer of Field B.

One possible explanation for these differences is the variation in tillage practices between the fields. Field A was subjected to conventional tillage to a depth of 25 cm, while Field B was managed with non-inversion tillage limited to 10 cm. Notably, the 0–25 cm sampling layer in Field A corresponds to the combined top (0–10 cm) and deeper (10–30 cm) sampling layers of Field B. As a result, the potential temporal effects in the top layer of Field A may have been mixed with soil from deeper layers, and, therefore, fewer temporal changes are observed. In contrast, the limited disturbance in Field B may have preserved distinct vertical stratification, allowing temporal effects to emerge more clearly in the top layer. While the impact of tillage practices was not specifically addressed in the experimental design, this observation has been confirmed in studies comparing conventional vs. reduced tillage, where different bacterial and fungal beta-diversities were observed (24).

In both fields, limited fluctuations in the fungal community were observed over time. This greater temporal stability observed for the fungal community has also been reported previously when studying the within-season variation (25). However, a recent 2-year study demonstrated that fungal communities, relative to bacterial communities, exhibited stronger temporal variation in the top soil across both years (26). This variation was associated with more distinct temperature and precipitation differences between the 2 years. Given that fungal communities generally have longer generation times than bacteria, leading to delayed responses to environmental fluctuations (27), it is possible that the shorter duration of our 1-year study was insufficient to capture the full extent of fungal temporal dynamics.

The within-field spatial variability was studied by comparing the different non-amended blocks of Field B in the top layer. We detected a strong effect on the fungal community but a weaker effect on the bacterial community. Controlling these spatial differences is important to ensure that sufficient samples have been obtained to detect temporal and management effects, given the high spatial variability of the microbial communities. These strong spatial effects are in accordance with other studies that compared between- (8, 28) and within-field variabilities (29). We hypothesize that the spatial distribution of the fungal community in Field B can be explained by two factors: (i) the historical background of the field and (ii) the varying sensitivity of fungi and bacteria to disturbances. Despite the field trial being operational under current management practices for more than a decade, there are still noticeable gradients in physicochemical soil properties (HWC) (L. Joos, unpublished data). Secondly, reduced tillage practices in the top layer of Field B have a more significant impact on bacteria than on fungi, which has been previously reported (30, 31). As a result, these practices lead to greater homogenization of the bacterial community in the top layer than for the fungal community. These data revealed pronounced spatial variability in the soil microbial community composition within one field. Therefore, it is opportune to create a well-thought experimental design that captures this variation by working with a block design that captures this variation (32).

The impact of the organic amendments, biochar or compost, had limited effect on the microbial communities when analyzed separately at each time point. However, it is worth noting that compost affected a few bacterial ASVs in March, May, and July, which was not observed when any other time point was analyzed alone. This shows the importance of the temporal effect and correct planning of the sampling moment. This underlines the significance of temporal effects and the importance of strategic sampling. Furthermore, when a global analysis was performed, assessing the average effect of amendments across all timepoints, a compost effect, but not a biochar effect, was observed at the bacterial family level (16). This result may reflect the different natures and effects of the two organic amendments. Biochar, which was applied only once, is a very stable but rather inert source of organic C, providing limited readily available C for microbial use. As a result, it contributes to a long-term increase in the soil organic C content over a longer period and minimal limited effects on the soil microbiome. In contrast, compost, which was applied yearly, provides additional C to the soil over a longer period. The initial higher HWC and HWP concentrations in the compost-amended soils, however, did not significantly affect the soil microbiome possibly because the concentration decreased toward the summer, and the effect might have been limited.

In our research, only limited changes were found before and after the drought period in July 2018. Additionally, chemical variables measured in the soil showed a trend over time, with either the lowest or highest concentrations occurring in the summer. This trend was not related to the applied treatments, as the same shifts were also observed in the control plots, although compost addition caused a lower increase in mineral nitrogen toward the summer. This observation confirms previous literature about carbon and nitrogen dynamics and might be related to the crops grown during that period (maize) and the exudates that were released (33).

### Temporal dynamics outweigh the effects of compost amendment in shaping microbial functions

Our study did not support the hypothesis that soil amendments would have a greater impact on microbial functions than on the taxonomic composition. From the PERMANOVA, no significant effect was found on functional categories, and no differentially expressed metabolic pathways were identified between non-amended and compost-amended soils. To our knowledge, no previous studies have conducted metatranscriptomics to understand the impact of compost amendment on soils. Somewhat comparable is a study that used a woody ash amendment (stable source of C with 12,000 kg ha^−1^) in agricultural soil, which revealed only minor functional transcript responses. In this study, the highest relative abundance of sequences was in the subsystems of protein metabolism, clustering-based carbohydrates, and amino acids and derivatives, which suggests that these are required functionalities in the microbial communities. This is also confirmed, as they are often reported as having the highest in relative abundance among different ecosystems, such as forests, grasslands, wastewaters, and Antarctic, Arctic, and tropical regions (20, 34–38).

In contrast, our study revealed that the functionality of the soil microbiome varied significantly depending on the season based on the PERMANOVA and differential expression analysis. During the dry and hot summer, we observed downregulations in transcripts related to nutrient availability and uptake potential of nutrients (e.g., metabolism and membrane transport subsystem categories [34, 35]), active growing of organisms (e.g., cell wall and capsule categories [34]), and disease suppression (e.g., virulence, disease, and defense categories [35]). These shifts suggest a reduction in microbial metabolic activity likely in response to environmental stressors, such as drought and heat, both of which occurred during the summer sampling period (Fig. S2).

An upregulation of osmotic stress response transcripts consistent with microbial adaptation to water limitation was observed. This was, however, accompanied by an unexpected downregulation of dormancy and sporulation-related genes (39). Considering that dormancy and sporulation are well-established microbial survival strategies under environmental stress, this pattern suggests a deviation from typical stress response mechanisms or potentially a limitation in the capacity for dormancy induction under environmental pressure (40, 41).

The summer period was also marked by increased expression in genes related to photosynthesis and CO_2_ uptake. This may suggest an opportunistic metabolic response by phototrophic microorganisms under elevated light and temperature conditions, potentially contributing to carbon cycling dynamics even under drought stress (42).

### The soil microbiome changes less in the deep layer than in the top soil layer

As expected, there were fewer spatiotemporal changes on the microbial community in the deep layer than in the top soil layer. The PLFA concentration in the deeper soil (10–30 cm) was approximately half of that in the top layer (0–10 cm), which is probably related to the lower amount of C in the deeper layer (43). This correlation between PLFA concentration and carbon became even more apparent in July when a decline in HWC and PLFA was observed, while an increase in the winter periods for both measurements was observed.

The different spatiotemporal patterns found for the bacterial community in this deeper layer compared to the top layer were unexpected. While strong temporal but limited spatial effects occurred in the top layer, the opposite response is found in the deeper layer where temporal variation became almost absent, while a clear spatial pattern became apparent. This pattern is likely correlated with the field history as observed for the fungal community, where blocks A/B and C/D correspond to the earlier outlines of the previous two field trials. The two field trials were subject to distinct management practices, resulting in potential heterogeneity within the fields. This heterogeneity is likely to persist due to the utilization of non-inverted tillage, which mainly impacts the top 10 cm of the soil. Additionally, the effects of precipitation and temperature primarily influence the top layer of the soil, increasing the heterogeneity between the two layers. These findings highlight the importance of understanding the impact of the depth at which samples are taken when conducting soil microbiome analyses. When multiple fields are being sampled and compared directly, it is crucial to select the most representative sampling depth for all fields to ensure accurate comparisons between them.

In conclusion, this study emphasizes the spatiotemporal dynamics of soil microbial communities within agricultural fields. We showed that spatiotemporal dynamics outweigh the effects of management and treatment practices and that the impact of these dynamics depends on the field, sampling depth, and studied community. We highlight the importance of microbial functions in combination with taxonomy to study the complexity of soil environments and microbial communities and underscore the need for a well-designed experimental and sampling scheme.

## MATERIALS AND METHODS

### Experimental field trials and sample collection

**Fig 8:**
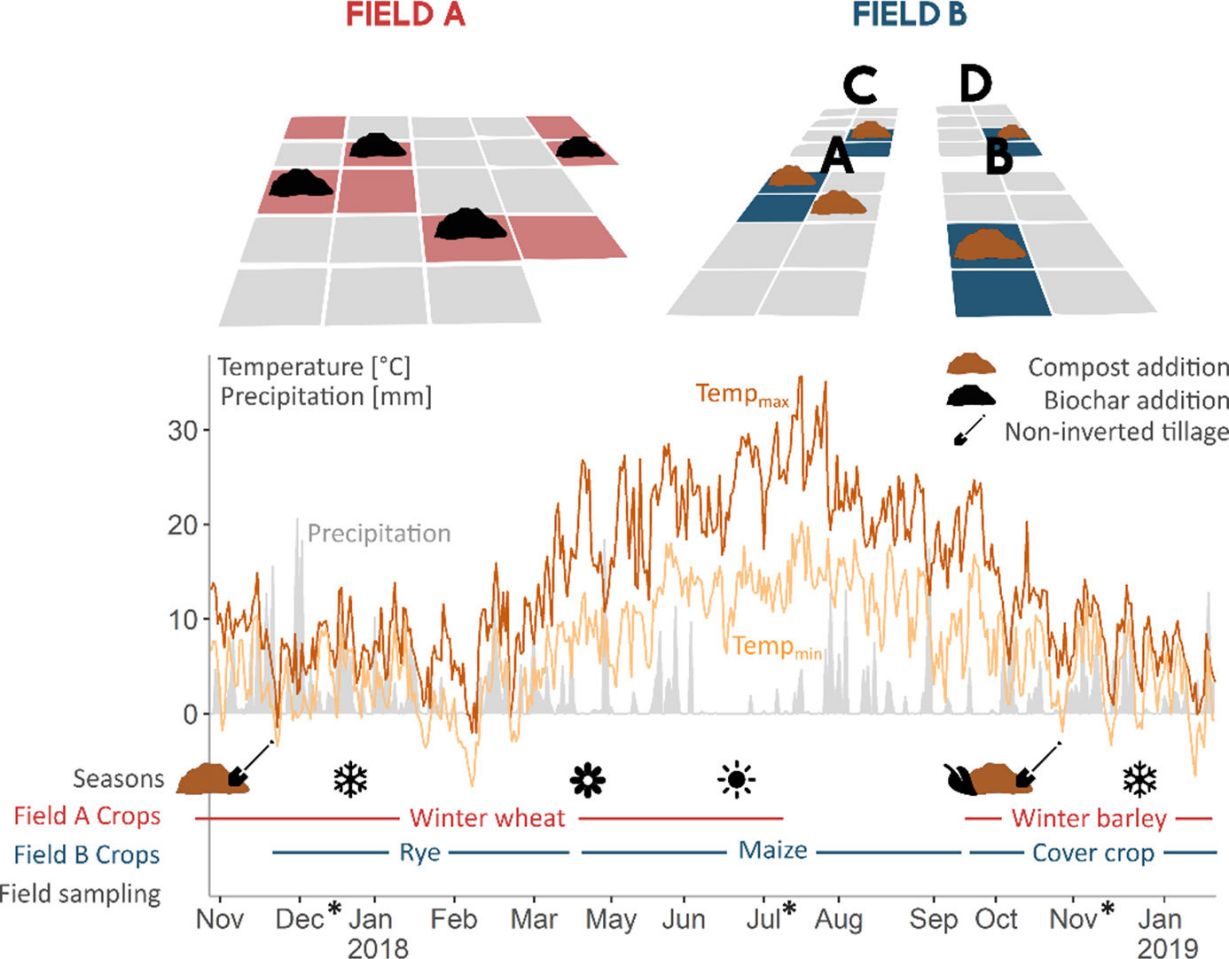
Experimental design and sampling strategy of the field trials. Field A was subjected to a randomized experimental design, while Field B had a strip-split block design. A subset of plots colored red for Field A and blue for Field B was sampled. These plots were either non-amended or amended with biochar (Field A) or compost (Field B). From November 2017 until January 2019, 13 sampling events were organized. The graph shows the range of minimum (light brown) to maximum (dark brown) ambient temperature and precipitation (gray) during this time. The seasons are marked by symbols. Climate is fully humid temperate with an extremely dry and warm summer period and cold and snowy winter periods during the sampling. Annual precipitation of 797 mm, a mean maximum temperature of 14°C, and mean minimum temperature of 6.8°C were recorded (KMI meteorological station, Melle). Field B received a yearly compost application in October, followed by non-inverted tillage (shovel symbol). The crops grown during the sampling period for Field A were winter wheat and winter barley, and those for Field B were rye, maize, and cover crops. Metabarcoding is done on all sampling events, while metatranscriptomics, hot-water extractable carbon (HWC), hot-water extractable phosphorus (HWP), and mineral nitrogen (N) were analyzed at the time points indicated with a *.

The soil samples were collected from two sandy loam field trials at ILVO (Flanders Research Institute for Agriculture, Fisheries and Food, Merelbeke, Belgium) (Fig. 8). Field A included four control plots and four biochar-treated plots (10,900 kg C ha^−1^ applied in 2012 and incorporated using conventional tillage to a depth of 25 cm with a moldboard plough) arranged in a completely randomized design (44). Field B consisted of four control plots and four compost-treated plots (2,000 kg C ha^−1^ y^−1^ applied annually) in a strip-split plot design (45). Additionally, for Field B, cattle slurry (170 kg N ha^−1^ y^−1^) was added, and non-inverted tillage with a cultivator (Actisol) to a depth of 10 cm was used to incorporate the amendments. Sampling was conducted every 5 weeks from November 2017 to January 2019 on the same day. Due to the suboptimal sampling quality, the first time point from Field B was excluded from the analysis, resulting in 13 sampling events for Field A and 12 for Field B. During the sampling period, Field A was planted with winter wheat (Oct. 2017–July 2018) and winter barley (from Sept. 2018), while Field B had rye (Nov. 2017–Mar. 2018), maize (Mar.–Sept. 2018), and cover crops (from Sept. 2018). No irrigation was used. Field A received mineral N fertilizer in spring based on crop needs. Field B received mineral N and K fertilizers alongside pig or cattle slurry (170 kg N ha⁻¹). Weed control was managed with herbicides, and insecticides were applied to winter barley (Biochar) following good agricultural practice. The samples were collected at depths of 0–25 cm for Field A and at depths of 0–10 and 10–30 cm for Field B to reflect the effects of the tillage practices on each field. Within each plot, eight soil cores were taken in an *X*-sampling pattern, meaning that cores were taken at four evenly spaced points along two diagonal lines that intersect at the center of the plot to create a composite sample. Further details of the sampling procedure are provided in reference 16. The samples were stored at −80°C for metatranscriptomic analysis, −20°C for chemical analyses, or freeze-dried before being stored at −20°C for PLFA and DNA extraction. For metabarcoding, all time points were analysed. For PLFA, chemical analyses, and metatranscriptomics, three time points (December 2017, July 2018, December 2018) were analyzed based on large differences in soil moisture content (Fig. S2). Detailed information on the chemical soil analyses and PLFA can be found in reference 46. Eighteen PLFAs served as biomarkers to profile the microbial community divided into six microbial groups: gram-negative (G^−^) bacteria, gram-positive (G^+^) bacteria, non-specific bacteria, actinomycetes, fungi, and arbuscular mycorrhizae.

### 16S rRNA and ITS2 gene amplicon sequencing

DNA extraction was performed on 250 mg soil using the DNeasy PowerSoil Kit according to the manufacturer’s protocol (Qiagen, Hilden, Germany). Illumina metabarcoding was done on the hypervariable V3–V4 (S-D-Bact-0341-b-S-17 forward and S-D-Bact-0785-a-A-21 reverse primers [47]) region of the 16S rRNA gene and the variable spaces of ITS2 gene (fITS7 bis-adapted forward and ITS4NGSr reverse [48]) for the bacterial and fungal communities, respectively, according to the described protocol by De Tender et al. (49). Libraries were sequenced by means of the Illumina MiSeq v3 technology (2 × 300 bp, paired-end) by Admera Health (South Plainfield, NJ, USA) with 30% PhiX DNA as spike-in (49). The bacterial and fungal samples were spread over multiple runs in a well-balanced design. Sequences were demultiplexed by the provider, and primers were removed by Trimmomatic (v0.32) for both the bacterial and fungal data sets (50). The DADA2 inference algorithm using R (v3.5.2) was used to process the demultiplexed sequence data following the protocol described in Joos et al. (31) for the bacterial and fungal communities (31). Taxonomy was assigned via the SILVA database for bacteria (v132) (51) and the UNITE database for fungi (v04.02.2020) (52) using the assignTaxonomy() function of DADA2 without taxonomic rank correction (53).

### RNA extraction and pre-processing of metatranscriptomics sequence data

Total microbial RNA (metatranscriptomics) was extracted from the no compost and compost samples from Field B taken in December 2017, July 2018, and December 2018. These time points were selected based on the most varying moisture content (Fig. S2) and resulted in 18 samples in total (number of biological replicates = 3).

An Eppendorf tube of 2 mL was filled with soil and immediately flash-frozen in liquid nitrogen until extraction with the RNA PowerSoil Total RNA Isolation Kit (Qiagen) as described in the manufacturer’s instructions. To remove remaining DNA from the samples, RNA was isolated with the Nucleospin RNA Kit (Machery-Nagel, Düren, Germany). Fluorometric quantification was done with the Quantifluor RNA System (Promega), and quality was checked by using a bio-analyzer (Agilent Technologies, Santa Clara, CA, USA) (Fig. S4). Library preparation and sequencing were provided by Admera Health (South Plainfield, NJ, USA). Briefly, the provider ribodepleted the libraries with NEBnext Ultra II with RiboZero rRNA Depletion Kit (Illumina, San Diego, CA, USA). Samples were sequenced on a HiSeq 3000 2 × 150 bp. Adaptors were removed by Trimmomatic (v0.39) using default settings (50). On average, 32,033,821 ± 3,541,130 reads were obtained per sample for the raw data (Table S1). The reads were merged and quality-filtered by the program, PEAR (v.0.9.11). A minimum length of 100 bp for reads, a minimum overlap size of 50 bp, and a quality score threshold of 25 were used for all sequences. After filtering and merging, 60% ± 6% of the raw reads were removed, retaining an average of 13,073,128 sequences. In the library preparation, ribosomal RNA (mainly from prokaryotes) was removed. The sortmeRNA pipeline (v4.2.0) was used as digital ribodepletion using the curated SILVA database for mapping (51, 54) (Table S2).

### Functional analysis of the metatranscriptomics data

The metatranscriptomes were compared to the SEED subsystem database in MG-RAST to retrieve functional information, resulting in three levels of resolution (levels 1–3) (55, 56), with level or subsystem 1 as the highest level of functional categorization. Filtered count tables on subsystem levels 1 and 3 were created. Reads that were assigned to a functional group should have an *e*-value of at least five, a percentage identity of 80, a minimal length of 15 amino acids, and a minimal abundance of 10. The statistical analysis of the data was performed with edgeR (57).

### Data analysis

The chemical, PLFA, and metabarcoding data were analyzed in R version 4.0.4, while the metatranscriptomics data were analyzed using version 4.1.1 (58). A combination of linear models (LM) and generalized linear models (GLM) was used depending on violations of normality and homoscedasticity to study the main effects of sampling time point and treatment (biochar, compost) and their potential interaction effect while correcting for variation introduced by performing the analysis or by the design (blocking). Generally, an LM was used for the chemical and PLFA analyses unless the assumptions of normal distribution of the residuals or homoscedasticity were violated, in which case a gamma-distributed GLM was applied. Each contrast was adjusted for multiple testing by using the Benjamini-Hochberg false discovery rate (BH-FDR) method (59). The applied models were used to assess multiple hypotheses of interest that are translated into linear combinations of the model parameters to test specific comparisons of interest (i.e., contrasts). Detailed information on the applied models, contrasts, associated hypotheses, and results of the statistical tests can be found in File S1.

The following mean models were applied:

Field A: g(*μ*) ~ time point * treatment + block_analysis_

Field B: g(*μ*) ~ time point * treatment * depth + block_analysis_ + block_design_

Field B_metatranscriptomics_: g(*μ*) ~ time point * treatment + block_design_

In these models, g(.) represents the link function, and *µ* is the modelled mean. The identity function was used for the LM or log link for GLM analyses unless specified otherwise. Both main and interaction effects are included in the models and indicated with “*.” Time point and treatment were included as a factor term. To account for variation introduced by the performed analyses of PLFA and metabarcoding, a block factor (block_analysis_) was added, indicated with a +. This information was not included in the other analyses (chemical and metatranscriptomics data). Additionally, a block factor for the field design of Field B (block_design_) was included.

After sequencing, the bacterial library depth was on average 43,996 reads (95% confidence interval [CI]: 39,803; 48,189) for Field A and 39,609 reads (95% CI: 37,810; 41,408) for Field B. The fungal library depth was 69,417 reads (95% CI 62,880; 75,954) for Field A and 69,319 reads (95% CI: 64,224; 74,414) for Field B. The metabarcoding data were processed by removing mitochondria and chloroplasts from the bacterial sequences. A technical filtering was applied by removing low-abundant sequences with less than two counts and present in at least three independent samples for both the bacterial and fungal sequences. After filtering, 475 bacterial ASVs in Field A, 687 bacterial ASVs in the top layer of Field B, and 350 bacterial ASVs in the deeper layer of Field B were found. In addition, 464 fungal ASVs for Field A and 373 fungal ASVs for both layers of Field B were identified. Diversity was calculated using the Shannon-Wiener index using the vegan package (60).

The bacterial and fungal community compositions of both fields were analyzed with PERMANOVA (60) on the dissimilarity matrix based on pairwise Bray-Curtis dissimilarity (60) indices and visualized by PCoA.

Differential abundance analyses on the bacterial and fungal metabarcoding data were conducted at ASV level to study the general effect of treatment, time, or spatial effect using edgeR (57). The ASV counts were modeled using nbGLM with a log link and a mean model as described for the (generalized) linear models. The data were normalized using offsets to correct for the effective library size using trimmed mean of *M* values (61). For each comparison, the differential abundance of specific contrasts was assessed with a likelihood ratio test. All contrasts are presented in File S1. Again, the BH-FDR was used to adjust for multiple testing of each contrast across the ASV levels, and the FDR was controlled at 5%. However, a more robust approach, namely, stage-wise testing, was adopted for the ASV level (62). In the screening stage, an omnibus test assesses the overall null hypothesis that an ASV is not differentially abundant in none of the comparisons vs. the alternative hypothesis that it is differentially abundant at least once. In the confirmation stage, individual hypotheses are assessed for ASVs that passed the screening stage. The fold changes of the DA confirmation stage hypotheses were then used to classify the screening stage ("overall") DA ASVs into three categories: (i) "up," upregulated in all significant hypotheses; (ii) "down," downregulated in all significant hypotheses; and (iii) "fluctuating," ASVs that increased in some hypotheses but decreased in others.

The microbial functions categorized on SEED subsystem level 3 (calculated by MG-RAST) were filtered by removing low-abundance sequences with fewer than three counts in at least three independent samples. A simplified model structure for the metatranscriptomics data was applied due to only analyzing the top layer and three of the four biological replicates. The subsystem 3 functional categories were analyzed with PERMANOVA and EdgeR after filtering as described above.

## Data Availability

The data sets supporting the conclusions of this article are available in the NCBI Sequence Read Archive under accession number PRJNA725989. The metatranscriptomics data are available in NCBI under BioProject number PRJNA931226 and in ENA under project number PRJEB26601.

## SUPPLEMENTAL MATERIAL

File S1 msystems.00112-25-s0001.xlsx Model contrasts and output of the models.

Supplemental material msystems.00112-25-s0002.docx Supplemental figures and tables.

ASM does not own the copyrights to Supplemental Material that may be linked to, or accessed through, an article. The authors have granted ASM a non-exclusive, world-wide license to publish the Supplemental Material files. Please contact the corresponding author directly for reuse.
