# Supplementary material for: Year-long, multiple-timepoint field studies show the importance of spatiotemporal dynamics and microbial functions in agricultural soil microbiomes
Source: mSystems. 2025 Jul 2;10(7):e00112-25. doi: 10.1128/msystems.00112-25 (PMC12282097; doi:10.1128/msystems.00112-25)
Supplement: Supplemental material — Supplemental figures and tables. [file msystems.00112-25-s0002.docx]

**Year-long, multiple time point field studies show the importance of spatiotemporal dynamics and microbial functions in agricultural soil microbiomes**

Lisa Joos^1,#^, Sarah Ommeslag^2^, Steve Baeyen^2^, Wouter Asselberg^2^, Koen Van Loo^2^, Lieven Clement^3^, Jane Debode^2^, Bart Vandecasteele^2^, Caroline De Tender^1^

Corresponding author^*^

^1^Ghent University, Department of Biochemistry and Microbiology, 9000 Ghent, Belgium

^2^Flanders Research Institute for Agriculture, Fisheries and Food (ILVO), Plant Sciences Unit, Merelbeke, Belgium

^3^Ghent University, Department of Applied Mathematics, Computer Science and Statistics, Ghent, Belgium


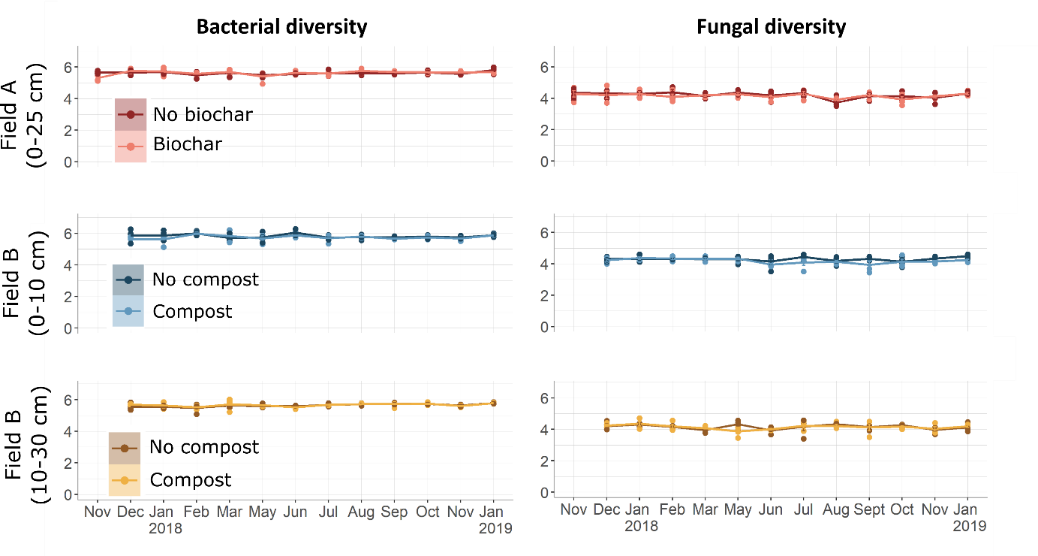


**Figure S1 | Time, treatment, and field-heterogeneity effects on the Shannon-Wiener diversity of the bacterial and fungal communities of Field A (red), Field B 0-10 cm (blue) and Field B 10-30 cm (yellow) over time.**

The different treatments are indicated by colour shade (light = non-amended, dark = amended). Dots represents the four biological replicates for each time point. No samples were collected for Field B in November 2017. No significant effects were found (GLM, P > 0.05).


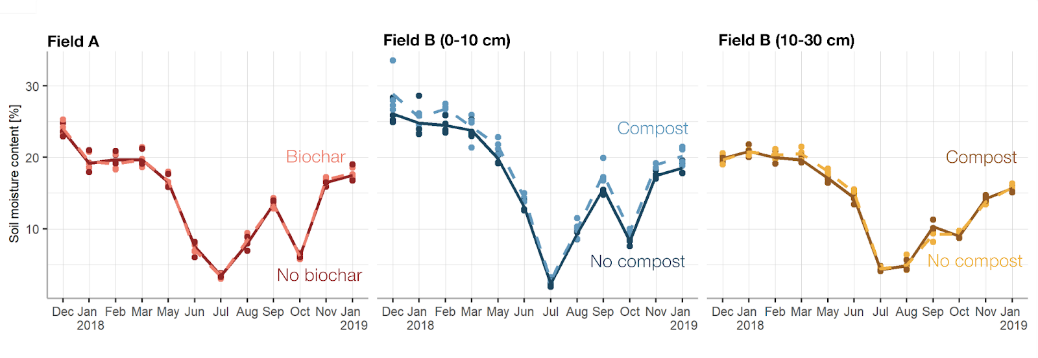


**Figure S2 | Soil moisture content of Field A (0-25 cm) and Field B (0-10 cm and 10-30 cm) over time.** Soil moisture content [%] was measured every sampling period. For each sampling moment, four biological replicates are shown ± standard error (SE), with the exception of the samples of June 2018 in Field B, as only three biological replicates were analyzed for the compost-treated soil in each depth layer. The darker full line represents the control for each field (either without biochar, or without compost), while the lighter dashed line represents the treatment for each field (either biochar or compost). This figure is adapted from (Joos et al., 2021).


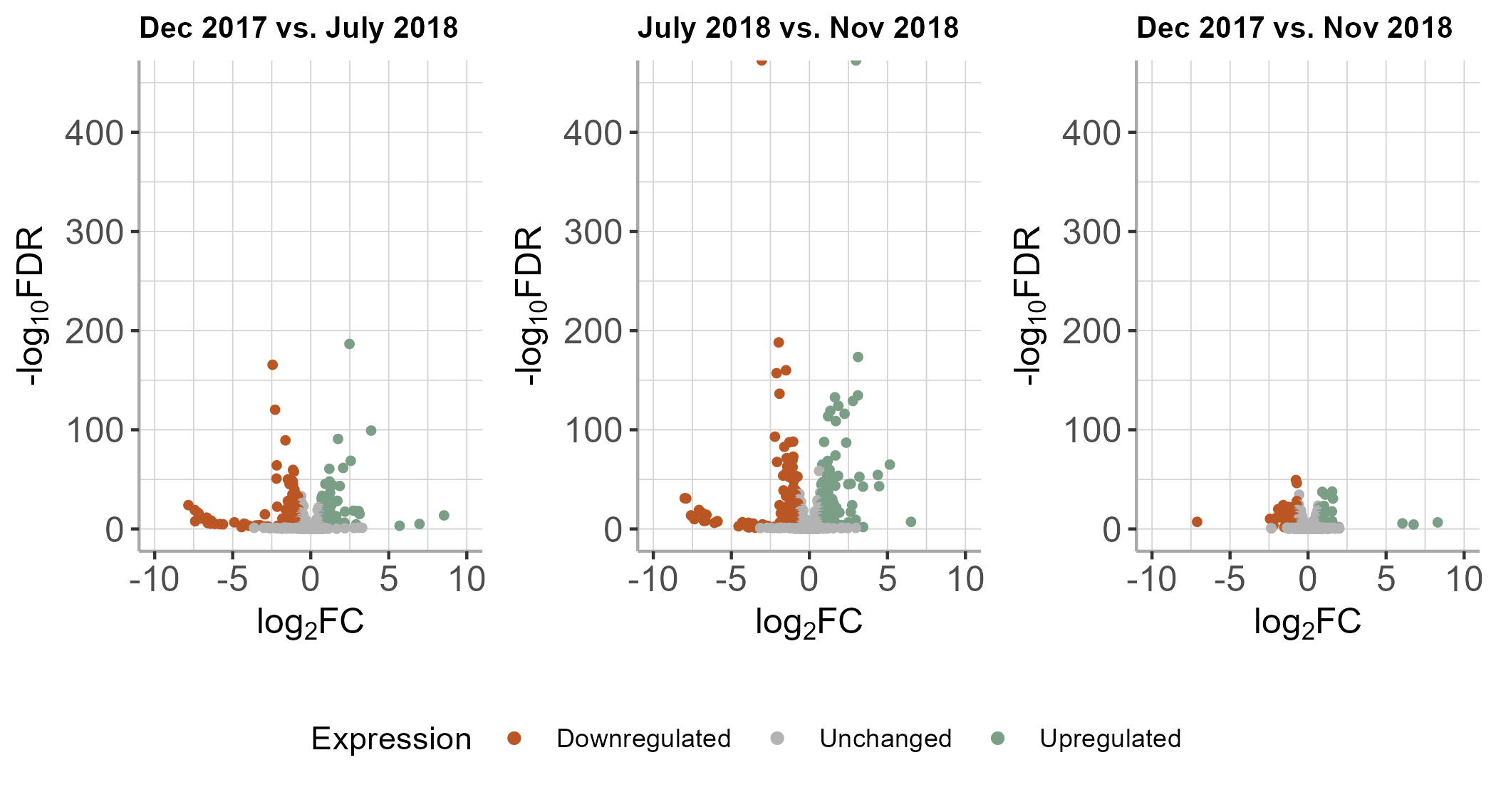


**Figure S3 | Differentially expressed (DE) level 3 categories (MG-RAST) for three time comparisons.**

In total 1,004 functional categories were found of which in total 543 were DE in Dec 2017 vs. July 2018 (up: 241, down: 302), 653 DE between July 2018 vs. Nov 2018 (up: 300, down: 353) and 467 DE between Dec 2017 vs. Nov 2018 (up: 229, down: 229). Colours indicate the up- or downregulated or unchanged functional categories.


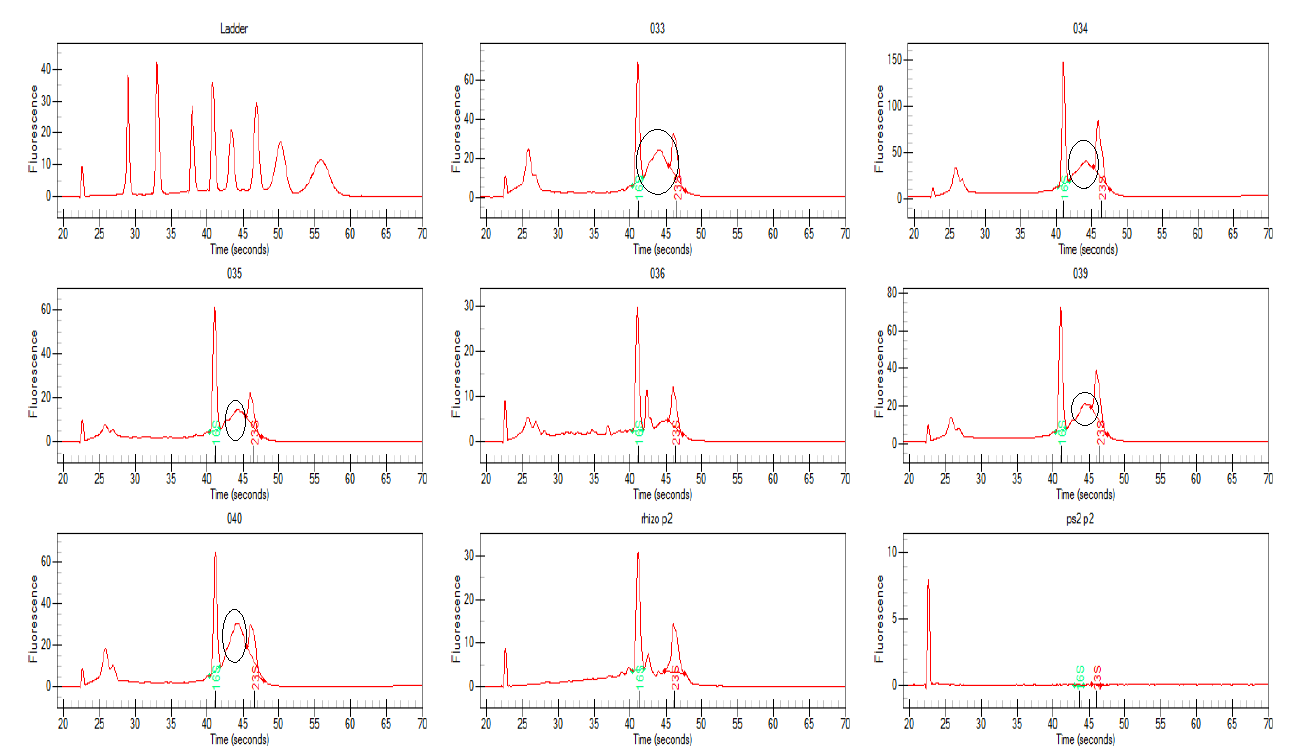


**Figure S4 | Quality control (bioanalyzer) of the RNA extracted with the RNA powersoil total RNA isolation kit and used for metatranscriptomics analysis.**

**Table S1 | Number of sequencing reads per sample on each step of the RNA-seq data preparation.**

The original number of sequencing reads (Raw reads), reads after quality control and trimming with trimmomatic, reads after merging the forward and reverse strand by Pear and the number of sequencing reads after removing all ribosomal RNA (rRNA) by applying sortmeRNA are shown for each sample. The last row shows an average value (± standard deviation for the raw reads) and the percentage decline compared to the original number of sequences. Samples are all from Field B and are uniquely identified by the combination of time point of sampling, treatment and biological replicates.

| Time point | Treatment | Bio rep | N° Raw reads | N° reads after trimming | N° Reads after merging |
| --- | --- | --- | --- | --- | --- |
| Dec17 | No compost | 1 | 37,735,386 | 25,928,061 | 18,834,316 |
|  |  | 2 | 33,477,276 | 21,852,291 | 16,781,154 |
|  |  | 3 | 35,385,072 | 22,137,303 | 16,298,787 |
|  | Compost | 1 | 33,004,043 | 20,485,494 | 16,487,688 |
|  |  | 2 | 31,989,070 | 20,949,056 | 16,333,235 |
|  |  | 3 | 35,018,787 | 21,604,116 | 16,550,321 |
| Jul18 | No compost | 1 | 30,893,257 | 14,824,799 | 10,656,126 |
|  |  | 2 | 25,389,274 | 12,803,583 | 8,979,875 |
|  |  | 3 | 33,667,945 | 18,395,944 | 11,628,328 |
|  | Compost | 1 | 23,592,532 | 11,783,323 | 8,416,401 |
|  |  | 2 | 33,353,329 | 17,660,394 | 11,521,301 |
|  |  | 3 | 27,464,825 | 14,838,816 | 9,797,810 |
| Nov18 | No compost | 1 | 32,701,244 | 18,439,906 | 12,063,520 |
|  |  | 2 | 33,056,643 | 18,274,538 | 12,588,012 |
|  |  | 3 | 30,288,324 | 16,105,066 | 11,101,499 |
|  | Compost | 1 | 35,324,695 | 19,411,012 | 12,776,993 |
|  |  | 2 | 32,374,480 | 18,013,113 | 12,444,354 |
|  |  | 3 | 31,892,606 | 17,652,157 | 12,056,585 |
| Mean |  |  | 32,03,821  (±3,541,130) | 17,697,832  (-43%) | 13,073,128  (-60%) |

Bio rep = biological replicates; N° = number

**Table S2 | Number of sequencing reads per sample after the analysis in MG-RAST.**

Input samples in MG-RAST were trimmed reads after merging (paired sequences). On average, 31% of the reads failed quality control (QC) (of which most were duplicate reads), 18% were annotated to an unknown protein, 18% was annotated to a known protein and 0.11% of the reads were identified as ribosomal RNA (rRNA).

| Time point | Treatment | Bio rep | Reads after merging | Reads failed QC | Unknown protein | Annotated Protein | rRNA |
| --- | --- | --- | --- | --- | --- | --- | --- |
| Dec17 | NC | 1 | 18,834,316 |  |  |  |  |
|  |  | 2 | 16,781,154 | 6,087,784 | 2,435,588 | 2,566,504 | 20,547 |
|  |  | 3 | 16,298,787 | 8,280,495 | 2,036,614 | 2,408,776 | 15,844 |
|  | C | 1 | 16,487,688 |  |  |  |  |
|  |  | 2 | 16,333,235 | 2,250,628 | 1,096,971 | 1,148,856 | 10,783 |
|  |  | 3 | 16,550,321 | 8,128,547 | 2,456,375 | 2,295,298 | 14,096 |
| Jul18 | NC | 1 | 10,656,126 | 2,423,855 | 1,807,357 | 2,053,242 | 16,208 |
|  |  | 2 | 8,979,875 | 1,932,402 | 1,605,104 | 1,742,064 | 16,203 |
|  |  | 3 | 11,628,328 | 2,045,324 | 1,977,534 | 2,038,028 | 23,101 |
|  | C | 1 | 8,416,401 | 1,625,080 | 1,456,073 | 1,501,877 | 10,281 |
|  |  | 2 | 11,521,301 | 2,041,356 | 1,901,083 | 2,007,483 | 18,953 |
|  |  | 3 | 9,797,810 | 1,997,834 | 1,676,845 | 1,733,677 | 14,732 |
| Nov18 | NC | 1 | 12,063,520 | 4,942,979 | 3,469,752 | 2,755,455 | 9,780 |
|  |  | 2 | 12,588,012 | 5,692,476 | 2,937,589 | 3,627,873 | 9,380 |
|  |  | 3 | 11,101,499 | 3,915,829 | 3,190,463 | 2,525,942 | 10,107 |
|  | C | 1 | 12,776,993 | 5,282,171 | 3,999,511 | 3,201,222 | 10,059 |
|  |  | 2 | 12,444,354 |  |  |  |  |
|  |  | 3 | 12,056,585 | 4,684,318 | 3,666,576 | 2,874,275 | 15,313 |
| Mean |  |  | 13,073,128 | 4,088,738 | 2,380,895 | 2,298,704 | 14,359 |
| % |  |  |  | 31 | 18 | 18 | 0.11 |
